# KIT over-expression by p55PIK-PI3K leads to Imatinib-resistance in patients with gastrointestinal stromal tumors

**DOI:** 10.18632/oncotarget.6011

**Published:** 2015-10-22

**Authors:** Senyan Lai, Guihua Wang, Xiaonian Cao, Xuelai Luo, Guoping Wang, Xianmin Xia, Junbo Hu, Jing Wang

**Affiliations:** ^1^ Department of Gastrointestinal Surgery Center, Tongji Hospital, Huazhong University of Science and Technology, Wuhan, 430030, China; ^2^ Department of Pathology, Tongji Hospital, Huazhong University of Science and Technology, Wuhan, 430030, China; ^3^ Department of Bioengineering, Hubei University of Technology, Wuhan, 430068, China; ^4^ Department of Immunology, Tongji Medical College, Huazhong University of Science and Technology, Wuhan, 430030, China

**Keywords:** p55PIK, Imatinib resistance, gastrointestinal stromal tumors

## Abstract

Imatinib is the first-line drug for gastrointestinal stromal tumors (GISTs), as mutated KIT is closely associated with the occurrence of GIST. However, Imatinib resistance (IMA-resistance) occurs inevitably in most GIST patients. Although the over-expression of KIT in GIST is one of the major factors contributing to IMA-resistance, the underlying mechanism is still unclear. In this study, we demonstrate that p55PIK, an isoform of phosphoinositide 3-kinase (PI3K), increases KIT expression, leading to IMA-resistance in GISTs by activating NF-κB signaling pathway. Furthermore, down-regulation of p55PIK significantly decreases KIT expression and re-sensitizes IMA-resistance-GIST cells to Imatinib *in vitro* and *in vivo*. Interestingly, the expression of both p55PIK and KIT proteins is significantly increased in tumor samples from IMA-resistance-GIST patients, suggesting that p55PIK up-regulation may be important for IMA-resistance in the clinical setting. Altogether, our data provide evidence that p55PIK-PI3K signaling can contribute to IMA-resistance in GIST by increasing KIT expression. Moreover, p55PIK may be a novel potential drug target for treating tumors that develop IMA-resistance.

## INTRODUCTION

Gastrointestinal stromal tumors (GISTs) are the most common mesenchymal tumors of the gastrointestinal tract. Gain-of-function mutations in KIT (a type III receptor tyrosine kinase operating in cell signal transduction in several cell types) or platelet-derived growth factor receptor (PDGFR) are found in most GISTs and these mutations typically are due to early events in GIST oncogenesis [1–3]. Imatinib (Glivec) is a selective small-molecule protein kinase inhibitor that is the standard therapy for GISTs [3–5]. Initial clinical responses to Imatinib are observed in about 80% of patients with metastatic GIST [1, 6, 7]. Although primary resistance to Imatinib only occurs in a minority of GIST patients, acquired resistance to Imatinib eventually occurs in almost all GIST patients during treatment. Currently, the mechanism(s) of Imatinib resistance (IMA-resistance) in GIST patients is still not well understood. Acquisition of new mutations in KIT often is observed in IMA-resistance-GIST patients [8, 9]; however, some reports also have shown that patients with IMA-resistance-GISTs do not have secondary KIT mutations [10, 11]. Several mechanisms of IMA-resistance have been proposed [11, 12], and it is likely that other, more complex, mechanisms are involved.

p55PIK is a regulatory subunit of Class I_A_ PI3K encoded by PIK3R3. It has no enzymatic activity, but is needed to recruit p110 subunits of PI3K to specific cellular targets and thus regulates the catalytic activity of PI3K [13, 14]. Recent reports have shown that p55PIK is over-expressed in many cancers, and has important roles in several oncogenic processes such as cell cycle regulation, cell growth, differentiation, metastasis, and angiogenesis [15–20]. Imatinib is widely-used in treating various leukemia patients, and can target Bcr-Abl, a fusion protein with enhanced protein kinese activity that plays a primary role in leukemia, particularly chronic myelogeous leukemia. We previously showed that blocking p55PIK-mediated signaling can improve Imatinib response in chronic myeloid leukemia (CML) [19], suggesting that p55PIK may play an important role in IMA-resistance.

In the present study, we showed that p55PIK is over-expressed in IMA-resistance-cell lines, GIST xenograft tumors, and clinical GIST specimens. Over-expression of p55PIK promoted IMA-resistance in Imatinib-sensitive GIST cells and xenograft tumors. Imatinib sensitivity was restored in IMA-resistance cells when p55PIK was knocked down using specific siRNAs. Importantly, p55PIK over-expression in Imatinib-sensitive GIST cells led to NF-κB pathway activation and induction of KIT expression. Additionally, when we used a p55PIK specific inhibitor, TAT-N24, IMA-resistance xenograft tumors were restored to Imatinib-sensitivity *in vivo* and *in vitro*. Collectively, our findings demonstrate that p55PIK plays an important role in IMA-resistance in GIST cells. Furthermore, p55PIK may be a useful prognostic marker for IMA-resistance in GIST and a potential therapeutic target for IMA-resistance-GIST.

## RESULTS

### Establishment of IMA-resistance-GIST cell line

To generate IMA-resistance-GIST cell lines, female nude mice were inoculated with GIST882 cells (2 × 10^6^ cells/0.1 ml saline), and 7 days after inoculation, were treated with Imatinib (100 mg/kg) administered by oral gavage daily for 3 weeks. Mice then were sacrificed and tumors (P1) removed, cut into small pieces, and re-implanted in new female nude mice. Mice bearing tumors (P1) were treated again with Imatinib (100 mg/kg) for 3 weeks. This procedure was repeated 6 times and tumors (P6) were removed from mice (Supplementary Figure S1A). The cells derived from these tumors were cultured, collected, and assessed for their sensitivity to Imatinib in comparison to the parental GIST882 cells by inoculating both cell lines in female nude mice that were then treated with Imatinib as described before. Parental GIST882 cells remained sensitive to Imatinib since the tumors in Imatinib-treated mice were smaller than tumors in control mice treated with vehicle alone (Supplementary Figure S1B). In contrast, cells derived from P6 tumors were relatively IMA-resistance since the size of tumors in mice treated with Imatinib was not significantly different than those in control mice (Supplementary Figure S1C). Also, cultured cells derived from P6 tumors were IMA-resistance when compared to parental GIST882 cells, and were named GIST882IR (Supplementary Figure S1D).

### Over-expression of KIT in GIST882IR cells

To better understand the mechanism of IMA-resistance, the expression level of KIT and mutations within the coding sequence of the KIT gene were examined in GIST882 and GIST882IR cells. Sequencing data confirmed that the KIT mutation, K642E, was present in GIST882IR and parental GIST882 cells [12]. However, no other KIT mutations were detected, suggesting that there were no secondary KIT mutations in GIST882IR cells (data not shown). Interestingly, Western blots showed increased KIT protein expression in GIST882IR cells compared to GIST882 cells (Supplementary Figure S2A). Additionally, RT-PCR showed that KIT mRNA was significantly up-regulated (Supplementary Figure S2B). To determine whether the transcription of KIT was enhanced in GIST882IR cells, a reporter plasmid (pGL-3) expressing luciferase under the control of a confirmed major promoter region in the KIT gene was constructed [24]. The vector was then transfected into GIST882 and GIST882IR cells and the luciferase activity of cell lysates was measured. GIST882IR cells displayed significantly increased luciferase activity compared to that in GIST882, suggesting that KIT transcription was increased in IMA-resistance-GIST cells (Supplementary Figure S2C). These data also suggested that the increase in KIT protein level in GIST882IR cells was due to increased KIT gene expression.

### Over-expression of p55PIK in GIST882IR cells and increased expression of p55PIK led to the IMA-resistance in GIST882 cells

**Figure 1:**
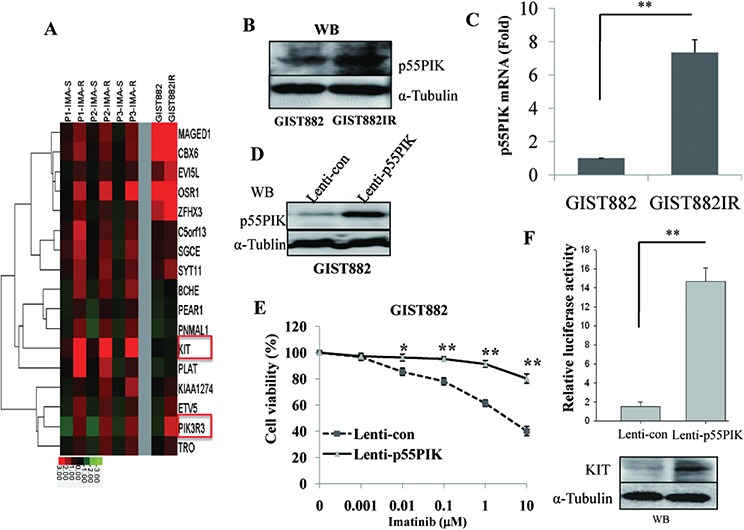
Over-expression of p55PIK in GIST882IR cells and IMA-resistance-GIST samples and increased expression of p55PIK led to the IMA-resistance in GIST882 cells **A.** Increased expression of KIT and p55PIK (PIK3R3) in GIST882IR and IMA-resistance-GIST samples. Gene expression profiles were analyzed in GIST882 and GIST882IR cells and primary (IMA-S) and recurrent (IMA-R) tumor samples from three GIST patients (P1, P2, P3) by microarray. **B.** Protein level of p55PIK in GIST882 and GIST882IR cells. **C.** mRNA level of p55PIK in GIST882 and GIST882IR cells. **D.** Increased p55PIK expression in GIST882 cells infected with lentivirus expressing p55PIK. GIST882 cells were infected with Lenti-p55PIK or control Lenti-con (MOI = 10) overnight. Cellular lysates were prepared 72 h after transfection and protein level of p55PIK was determined by Western blotting. **E.** IMA-resistance in GIST882 cells over-expressing p55PIK. GIST882 cells were infected with Lenti-p55PIK or Lenti-con overnight and treated with various concentration of Imatinib for 72 h. Cell viability was measured. *p < 0.05; **p < 0.01. **F.** Over-expression of p55PIK increased expression of KIT in GIST882 cells. Cultured GIST882 cells were infected with Lenti-p55PIK or Lenti-con overnight, then transfected with KIT promoter reporter plasmids. Cellular lysates were prepared 48 h after transfection, the protein level of KIT and the luciferase activity was determined. **p < 0.01.

To elucidate the mechanism of IMA-resistance in GIST cells, gene expression profiles were examined in GIST882 and GIST882IR cells as well as primary and recurrent tumor samples from three IMA-resistance patients undergone two surgical operations for GIST. These patients were treated with Imatinib after initial surgery until they developed IMA-resistance and required a second operation. Gene expression profiles of GIST tissues obtained from primary and secondary surgical resections were compared to those of GIST882IR and GIST882 cells. 143 genes were identified that were differentially up-regulated by greater than 2-fold in the IMA-resistance-patient tumors and GIST882IR cells (Figure 1A and data not shown). Of note, the KIT mRNA expression was increased in GIST882IR cells and the three IMA-resistance-GIST samples. Interestingly, PIK3R3, the gene that encodes p55PIK, was among the genes that were significantly up-regulated and thus was further characterized. Consistent with these data, p55PIK mRNA and protein expression were increased in GIST882IR cells (Figure 1B, 1C).

To identify the role of p55PIK in IMA-resistance, we over-expressed p55PIK in GIST882 cells and examined its effects on sensitivity to Imatinib as well as KIT mRNA expression. Accordingly, we infected GIST882 cells with a lentivirus construct expressing p55PIK (Lenti-p55PIK) or lentivirus control (Lenti-con) overnight. Cells were then collected 72 h after infection, and the protein expression level of p55PIK was determined. We observed that there was significantly increased p55PIK in GIST882 cells infected with Lenti-p55PIK compared to control cells infected with Lenti-con (Figure 1D). We then assessed Imatinib sensitivity in these cells, and found that GIST882 cells over-expressing p55PIK were more IMA-resistance than cells infected with control Lenti-con constructs (Figure 1E). KIT protein level and KIT promoter activity were significantly increased in GIST882 cells over-expressing p55PIK (Figure 1F), suggesting that a pathway mediated by p55PIK could potentially confer IMA-resistance by inducing KIT expression.

### p55PIK enhanced the expression of KIT by activating NF-κB signaling in GIST882IR cells

**Figure 2:**
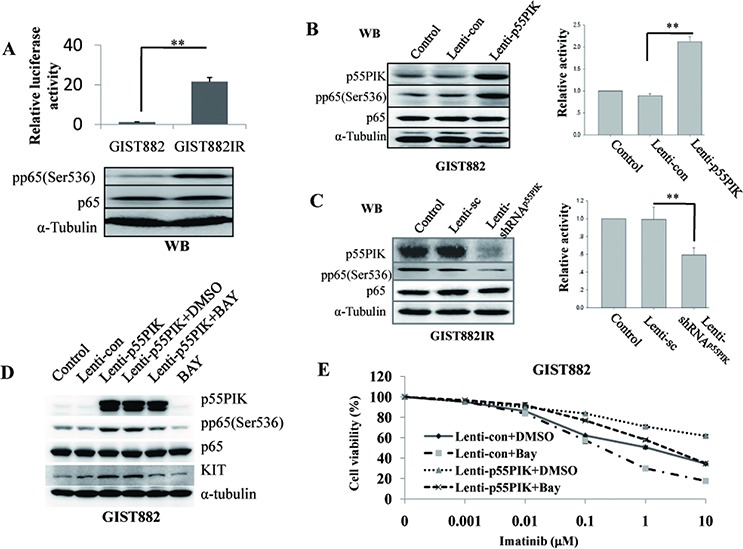
p55PIK regulation on KIT expression was mediated by NF-κB signaling **A.** Increased activation of NF-κB signaling in IMA-resistance-GIST cells. (Up) Enhanced NF-κB transcription activity in GIST882IR cells. Plasmid constructs containing a major NF-κB promoter element (pGL3-NF-κB element reporter) were used to transfect GIST882 and GIST882IR cells. Cell lysates were prepared 48 h after transfection and the relative luciferase activity in GIST882 and GIST882IR cells was measured. **p < 0.01; (Down) Increased NF-κB p65 phosphorylation in GIST882IR cells. The expression of p65 and the phosphorylation of p65 (pp65(Ser536)) in GIST882 and GIST882IR cells were determined by Western blotting. **B.** p55PIK induced the NF-κB activation in GIST882 cells. Cultured GIST882 cells were infected with Lenti-p55PIK or Lenti-con overnight and were transfected with pGL3-NF-κB element reporter plasmid. The cell lysates were prepared and analyzed. (Left) Western blotting of p65, pp65 (Ser536) and p55PIK expression levels in GIST882 cells infected with Lenti-p55PIK or Lenti-con. (Right) Relative luciferase activity of pGL3-NF-κB element reporter in GIST882 cells infected with Lenti-p55PIK or Lenti-con lentivirus. **p < 0.01. **C.** Decreased p55PIK expression inhibited the NF-κB activation in GIST882IR cells. Cultured GIST882IR cells were infected with Lenti-shRNAp55PIK or Lenti-sc overnight and were transfected with pGL3-NF-κB element reporter plasmid. The cell lysates were prepared and analyzed. (Left) Western blotting of p65, pp65 (Ser536) and p55PIK expression levels in GIST882IR cells infected with Lenti-shRNAp55PIK or Lenti-sc. (Right) Relative luciferase activity of pGL3-NF-κB element reporter in GIST882IR cells infected with Lenti-shRNAp55PIK or Lenti-sc. **p < 0.01. **D.** NF-κB inhibitor Bay11–7082 (BAY) blocked the expression of KIT and phosphsorylation of NF-κB p65 (pp65(Ser536)) induced by p55PIK. Cultured GIST882 cells were infected with Lenti-p55PIK or Lenti-con overnight and were incubated with BAY (final concentration: 5 μM) or solvent (DMSO) for 72 h. The cell lysates were prepared and analyzed. The expression of KIT, p55PIK and the phosphorylation of NF-κB p65 (pp65(Ser536)) were examined by Western blotting. **E.** NF-κB inhibitor Bay11–7082 (BAY) potentiated the inhibitory effects of Imatinib on GIST882 proliferation with or without the over-expression of p55PIK. Cultured GIST882 cells were infected with Lenti-p55PIK or Lenti-con overnight and were incubated with various concentrations of Imatinb in the presence of BAY (final concentration: 5 μM) or solvent (DMSO) for 72 h. Cell viability was determined.

NF-κB signaling occupies a central position within the regulatory network that controls KIT expression [25]. Previously, we found that p55PIK activated the NF-κB pathway in several cancer lines [20], so we examined whether p55PIK regulated KIT expression in GIST via the NF-κB signaling pathway. For this purpose, we constructed a luciferase reporter under the control of the major NF-κB transcription element [26] in pGL3 plasmid and determined the effects of p55PIK on NF-κB-mediated transcription in GIST cells. NF-κB promoter activity was significantly increased and phosphorylation of NF-κB p65 (Ser536) was increased in GIST882IR cells compared to GIST882 cells (Figure 2A). Furthermore, over-expression of p55PIK in GIST882 cells increased both the phosphorylation of NF-κB p65 (Ser536) and NF-κB promoter activity (Figure 2B), whereas knockdown of p55PIK in GIST882IR cells decreased the phosphorylation of p65 (Ser536) and NF-κB promoter activity (Figure 2C). Next, we used a NF-κB pathway inhibitor, BAY11–7082, to examine the role of NF-κB-mediated pathways on KIT expression. BAY11–7082 inhibited the phosphorylation of p65 (Ser536) and the expression of KIT in GIST882 cells, indicating that NF-κB regulated the expression of KIT (Figure 2D). Furthermore, Bay11–7082 prevented the increase in KIT expression due to p55PIK over-expression in GIST882 cells, suggesting that regulation of KIT expression by p55PIK likely depended on activation of the NF-κB pathway (Figure 2D). We next examined the effects of inhibiting NF-κB on Imatinib sensitivity in GIST882 cells, and found the addition of Bay11–7082 could potentiate the Imatinib inhibitory effects on proliferation in GIST882 cells with or without the over-expression of p55PIK, indicating that the inhibition of NF-κB restored the sensitivity of IMA-resistance-GIST cells to Imatinib (Figure 2E).

### Down-regulation of p55PIK or blocking NF-κB signaling led to decreased KIT and re-sensitized GIST882IR cells to imatinib

**Figure 3:**
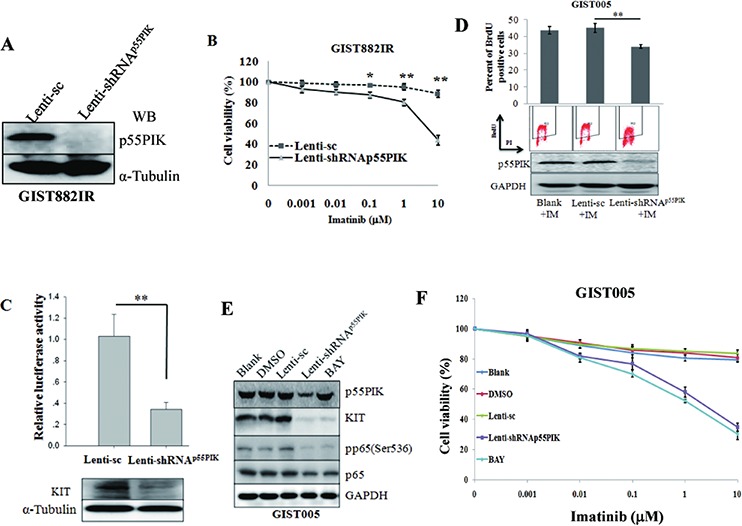
Down-regulation of p55PIK or blocking NF-κB signaling led to decreased KIT and re-sensitized GIST882IR cells to Imatinib **A.** Decreased p55PIK expression in GIST882IR cells infected with lentivirus expressing shRNA against p55PIK. GIST882IR cells were infected with Lenti-shRNA^p55PIK^ or control Lenti-sc (MOI = 10) overnight. Cell lysates were prepared and protein level of p55PIK was determined by Western blotting. **B.** Restoration of Imatinib sensitivity in GIST882IR cells infected with Lenti-shRNA^p55PIK^. GIST882IR cells were infected with Lenti-shRNA^p55PIK^ or Lenti-sc overnight and treated with various concentration of Imatinib for 72 h. Cell viability was measured. *p < 0.05; **p < 0.01. **C.** Down-regulation of p55PIK decreased expression of KIT in GIST882IR cells. Cultured GIST882IR cells were infected with Lenti-shRNA^p55PIK^ or Lenti-sc, then transfected with KIT promoter reporter plasmids. Cellular lysates were prepared 48 h after transfection, the protein level of KIT and the luciferase activity was determined. **D.** BrdU incorporation assay showed that down-regulation of p55PIK re-sensitized GIST005 cells to Imatinib. Cultured GIST005 cells were infected with Lenti-shRNA^p55PIK^ or Lenti-sc, after transfection for 72 h, Imatinib (10 μmol) was added in cultured cells, BrdU incorporation was checked at 72 h after Imatinib was added. **E.** Down-regulation of p55PIK or blocking NF-κB signaling led to decreased KIT in GIST005 cells. Cultured GIST005 cells were infected with Lenti-shRNA^p55PIK^, Lenti-sc or NF-κB signaling inhibitor Bay11–7082 (BAY) (final concentration: 5 μM). Cellular lysates were prepared 48 h after transfection or BAY treated, The expression of KIT, p55PIK and the phosphorylation of NF-κB p65 (pp65(Ser536)) were examined by Western blotting. **F.** Restoration of Imatinib sensitivity in GIST005 cells infected with Lenti-shRNA^p55PIK^ or using NF-κB signaling inhibitor Bay11–7082 (BAY). GIST005 cells were infected with Lenti-shRNA^p55PIK^ or Lenti-sc overnight and were incubated with various concentrations of Imatinb in the presence of BAY (final concentration: 5 μM) or solvent (DMSO) for 72 h. Cell viability was determined. *p < 0.05; **p < 0.01.

Next, we knocked down p55PIK expression in GIST882IR cells by using a lentivirus construct expressing shRNA against p55PIK (Lenti-shRNA^p55PIK^) and a lentivirus expressing scrambled shRNA (Lenti-sc) as vector control. p55PIK protein expression was significantly less in GIST882IR cells infected with Lenti-shRNA^p55PIK^ than cells infected with Lenti-sc (Figure 3A). We then assessed Imatinib sensitivity in these cells and found that knockdown of p55PIK in GIST882IR cells re-sensitized them to Imatinib (Figure 3B). Moreover, KIT protein and KIT promoter transcriptional activity were decreased when p55PIK was knocked down in GIST882IR cells (Figure 3C).

To further confirm down-regulation of p55PIK led to decreased KIT and re-sensitized GIST882IR cells to Imatinib, we knocked down p55PIK in primary cultured GIST cells GIST005, which was established from a secondary Imatinib-resistance GIST patient. BrdU incorporation assay showed that down-regulation of p55PIK re-sensitized GIST005 cells to Imatinib (Figure 3D). Moreover, similar to the results seen in GIST882IR cells, down-regulation of p55PIK led to decreased phosphorylation of NF-κB p65 (Ser536) and decreased KIT expression in GIST005 cells (Figure 3E). Knockdown of p55PIK expression or using NF-κB inhibitorBay11–7082 also led to Imatinib re-sensitized in GIST005 cells (Figure 3F). These findings suggested that p55PIK modulated KIT expression and inhibition of p55PIK-mediated signaling re-sensitized IMA-resistance-GIST cells to Imatinib.

### Over-expression of p55PIK increased the expression of KIT and led to the IMA-resistance in GIST-T1 and primary cultured GIST cells

We then examined the effects of p55PIK over-expression on Imatinib sensitivity and downstream signaling in another GIST cell line, GIST-T1 [21]. Similar to the results seen in GIST882 cells, over-expression of p55PIK led to phosphorylation of NF-κB p65 (Ser536) and increased KIT expression in GIST-T1 cells (Supplementary Figure S3A). Increased p55PIK expression also led to IMA-resistance in GIST-T1 cells (Supplementary Figure S3B).

To further confirm the effects of p55PIK over-expression on Imatinib sensitivity and downstream signaling in GIST, we overexpressed p55PIK in primary cultured GIST cells GIST002, which was established from an Imatinib sensitive GIST. Similar to the results seen in GIST882 and GIST-T1 cells, over-expression of p55PIK led to increased phosphorylation of NF-κB p65 (Ser536) and increased KIT expression in GIST002 cells (Supplementary Figure S3C). Increased p55PIK expression also led to IMA-resistance in GIST002 cells (Supplementary Figure S3D). Collectively, all these data suggesting that co-occurrence of p55PIK and KIT over-expression may be a feature of IMA-resistance in GIST.

### Down-regulation of p55PIK re-sensitized the IMA-resistance-GIST to imatinib *in vivo*

**Figure 4:**
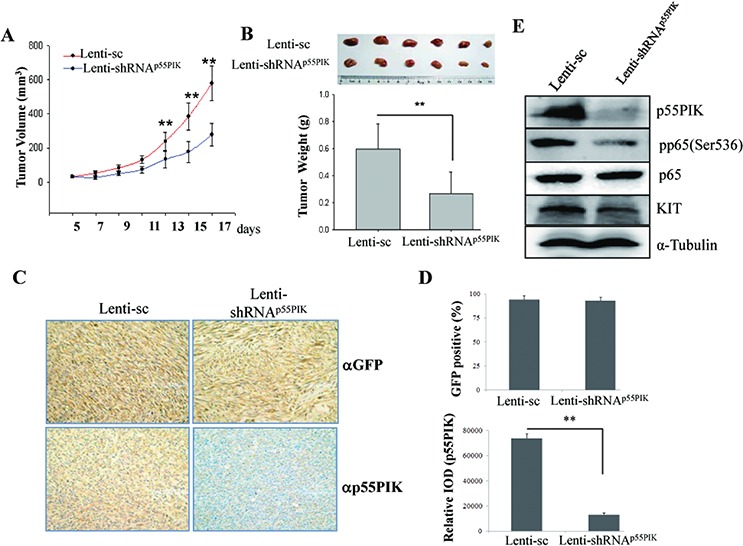
Down-regulation of p55PIK re-sensitized GIST882IR tumors to Imatinib *in vivo* GIST882IR cells were resuspended in culture medium (2 × 107 cells/ml) and injected subcutaneously into athymic nude mice (100 μl/tumor). At day 7 after inoculation Imatinib was given by intragastric administration (100 mg/kg) daily as well as Lenti-sc or Lenti-shRNA^p55PIK^ (10^10^ tu/20 μl) was injected into tumors every 3 days. **A.** Down-regulation of p55PIK decreased the tumor volume of GIST882IR *in vivo*. Tumor volume was measured every 2 days. *p < 0.05; **p < 0.01. **B.** Down-regulation of p55PIK potentiated the inhibitory effects of Imatinib on the tumor growth of GIST882IR cells *in vivo*. Tumors injected with Lenti-sc or Lenti-shRNA^p55PIK^ were weighed and analyzed. **C.** Immuno-histochemical (IHC) analysis of tumor samples using anti-GFP and anti-p55PIK antibodies. Representative images from IHC analysis detecting GFP signal (upper panel) or p55PIK signal (lower panel) shown. **D.** Upper panel: the percentage of GFP-positive cells in tumor samples was determined and used as an indicator to show the lentivirus transfection efficiency from intratumoral injection (mean ± SE (*n* = 3)). Lower panel: IHC quantification of p55PIK expression. The expression of p55PIK in Lenti-sc or Lenti-shRNA^p55PIK^-treated tumor samples were examined by IHC and the intensity of signal (Relative IOD) was determined and analyzed. **p < 0.01. **E.** Down-regulation of p55PIK decreased the expression of KIT and phosphorylation of NF-κB p65 in xenograft tumors. The expression of p55PIK, p65, pp65(Ser536) and KIT in tumors from GIST882IR cells injected with Lenti-sc or Lenti-shRNA^p55PIK^ was examined by Western blotting.

Next, we examined the effects of down-regulation of p55PIK on re-sensitization of IMA-resistance-GIST to Imatinib *in vivo*. GIST882IR cells were inoculated in nude mice. At day 7 after inoculation, Imatinib was administered by oral gavage daily and either lentivirus expressing shRNA against p55PIK (Lenti-shRNA^p55PIK^) or control lentivirus (Lenti-sc) (10^10^ tu/20 μl) was directly injected into tumors every 3 days [23]. Tumor volumes were measured every two days and mice then were sacrificed after 17 days, and their tumors were collected and weighed. The tumors that received Lenti-shRNA^p55PIK^ injection were smaller than control tumors that received Lenti-sc (0.28 ± 0.07cm^3^ vs. 0.58 ± 0.1cm^3^ at day 17, Figure 4A) and weighed significantly less than the control tumors (0.27 ± 0.16g vs. 0.60 ± 0.19g, Figure 4B). As lentivirus vector contained the cDNA sequence encoding GFP and the percentage of GFP-positive cells was used as an indicator to determine the lentivirus transfection efficiency from intratumoral injection. Results from Immuno-histochemical analysis showed that the transfection efficiency in tumor samples was 94% and 92% in Lenti-sc and Lenti-shRNA^p55PIK^-treated tumors, respectively (Figure 4C, 4D, upper panels). Furthermore, the expression of p55PIK was significantly decreased in tumors receiving Lenti-shRNA^p55PIK^ injections (Figure 4C, 4D, lower panels). The expression of p55PIK, p65, KIT and p65 phosphorylation (pp65(Ser536)) in the tumors were examined by Western blotting. Results showed that expression of p55PIK and KIT protein as well as NF-κB p65 phosphorylation (pp65(Ser536)) were decreased in tumors receiving Lenti-shRNA^p55PIK^, indicating that down-regulation of p55PIK not only led to the re-sensitization of IMA-resistance-GIST cells to Imatinib but also inhibited the NF-κB signaling and KIT expression (Figure 4E).

### Blockade of p55PIK signaling restored the sensitivity of IMA-resistance-GIST to imatinib *in vivo*

**Figure 5:**
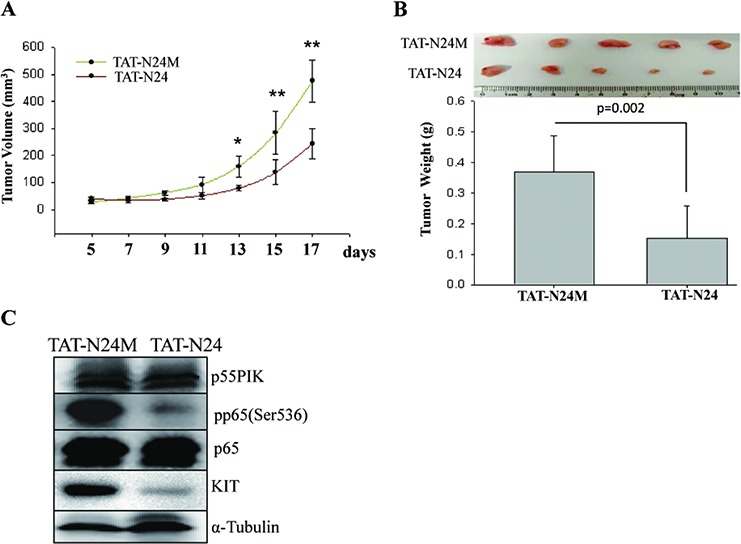
Pharmacologic inhibition of p55PIK by TAT-N24 restored Imatinib sensitivity in IMA-resistance-GIST tumors GIST882IR cells were injected subcutaneously into athymic nude mice. At day 7 after inoculation Imatinib (100 mg/kg) was given by intragastric administration. Additionally, mice were given TAT-N24 or TAT-N24M via tail vein as described previously^20^. **A.** TAT-N24 increased the inhibitory effects of Imatinib on the IMA-resistance-GIST growth *in vivo*. Tumor volume was measured every 2 days. *p < 0.05; **p < 0.01. **B.** TAT-N24 potentiated the inhibitory effects of Imatinib on the IMA-resistance-GIST growth *in vivo*. Tumors collected from mice receiving TAT-N24 or control TAT-N24M were weighed and analyzed. **C.** TAT-N24 inhibited the expression of KIT and phosphorylation of NF-κB p65 (pp65(Ser536)) in IMA-resistance-GIST xenografts. The expression of p55PIK, p65, KIT and the phosphorylation of p65 (pp65(Ser536)) in tumor xenografts were determined by Western blotting.

We previously developed a fusion protein (TAT-N24) that inhibited p55PIK signaling pathways [19]. We thus examined the effects of TAT-N24 on tumor growth of IMA-resistance-GIST in the presence of Imatinib *in vivo*. GIST882IR cells were inoculated in nude mice and Imatinib (100 mg/kg) was orally administered daily for 7 days after inoculation. TAT-N24 (80 mg/kg) or control peptide (TAT-N24M) then was injected via tail vein every 2 days for 20 days. Mice then were sacrificed and the tumors were collected and weighed. The tumors receiving TAT-N24 were smaller (0.24 ± 0.06 cm^3^ vs. 0.47 ± 0.08 cm^3^, Figure 5A) and weighed significantly less than the tumors receiving control TAT-N24M peptide (0.15 ± 0.12g vs. 0.37 ± 0.12g, Figure 5B). The expressions of p55PIK, p65, KIT and phosphorylation of p65 (Ser536) were also examined (Figure 5C). Results showed that expression of p55PIK was not changed; however, the expression of KIT and NF-κB pp65 (Ser536) was decreased, indicating that p55PIK signaling blockade inhibited the NF-κB signaling and KIT expression, which in turn, led to inhibition of tumor growth of IMA-resistance-GIST *in vivo*.

### Over-expression of KIT and p55PIK and increased activation of NF-κB in tumor samples from GIST patients with IMA-resistance

**Figure 6:**
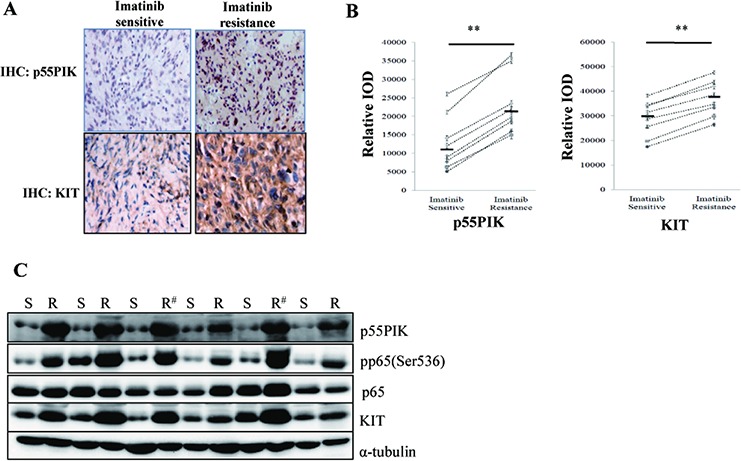
Over-expression of KIT and p55PIK and NF-κB activation in tumor samples from IMA-resistance-GIST patients **A.** Increased expression of KIT and p55PIK in IMA-resistance-GIST patients. The expression of p55PIK and KIT were detected by immunohistochemistry (IHC) in primary (Imatinib-sensitive) and recurrent (Imatinib-resistant) tumor samples from GIST patients. **B.** IHC quantification of p55PIK and KIT expression in primary (Imatinib-sensitive) and recurrent (Imatinib-resistant) tumor samples from GIST patients. The expression of KIT and p55PIK in primary and recurrent tumor samples from 8 IMA-resistance-GIST patients were examined by IHC and the intensity of signal (Relative IOD) was determined and analyzed. **p < 0.01. **C.** Increased expression of KIT, p55PIK and enhanced phosphorylation of NF-κB p65 in IMA-resistance-GIST samples from patients. The primary (S) and recurrent (R) tumor samples from 6 GIST patients were analyzed in Western blotting using various antibodies against KIT, p55PIK, p65, pp65(Ser536) and α-tubulin (# indicating the samples containing the secondary KIT mutations in recurrent tumor samples).

Next, we examined whether the results obtained from these *in vitro* and *in vivo* studies were clinically relevant in GIST patients. We analyzed mutations of the KIT gene, expression of p55PIK and KIT, and phosphorylation of NF-κB p65 (Ser536) in surgically-dissected tumor samples before and after Imatinib treatment from 8 GIST patients (the initial Imatinib treatment of these patients are positive response, detailed information about the patients are shown in Supplementary Table S1). All patients received Imatinib treatment after their first surgery and developed IMA-resistance and recurrence of GIST prior to their second surgery. Among the 8 patients, second mutations were observed in IMA-resistance-GIST samples in two patients (No. 3 and 5) whereas no new mutations were found in the other IMA-resistance samples. Significantly, over-expression of p55PIK and KIT was observed in all IMA-resistance samples (a patient tumor sample, pre- and post-treatment) (Figure 6A). The histochemical staining of p55PIK and KIT proteins in tumor samples was quantitatively determined and showed that the expression of both p55PIK and KIT were strongly increased in IMA-resistance tumor samples (Figure 6B). p55PIK and KIT protein expression, and NF-κB p65 (Ser536) phosphorylation were also strongly increased, even in IMA-resistance tumor samples from GIST patients with secondary mutations (No. 3 and 5, marked with #) (Figure 6C). These data confirmed our earlier *in vitro* and *in vivo* studies, and strongly suggest that over-expression of p55PIK likely contributes to IMA-resistance in GIST patients

## DISCUSSION

GIST is the most common human sarcoma and has been a primary model for targeted molecular therapy. GIST growth critically depends on oncogenic KIT signaling so tumors often respond initially to the tyrosine kinase inhibitor, imatinib. However, it now is clear that GISTs in patients who initially respond to Imatinib eventually develop IMA-resistance. It has been speculated that IMA-resistance may develop due to multiple factors, including drug bioavailability, treatment compliance, other KIT–independent genetic changes, and secondary KIT mutations [8, 9, 12, 27, 28]; however, currently the mechanism(s) for IMA-resistance in GIST is not fully understood. In this study, we examined the expression of inter-related proteins and signaling pathways in an established IMA-resistance variant from GIST882 cells and collected primary Imatinib-sensitive and IMA-resistance tumors from GIST patients to elucidate the mechanism of IMA-resistance in GIST. Here, we present evidence for a novel mechanism for IMA-resistance in GIST that involves increased KIT expression that is mediated by p55PIK-PI3K activation of NF-κB, a major regulator of KIT expression.

It currently is believed that the most common mechanism of acquired IMA-resistance in GIST patients is through secondary KIT mutations that disrupt Imatinib binding to KIT. Our results suggest that over-expression of KIT protein also is an important mechanism for IMA-resistance in GIST. Evidence supporting this notion are: First, recurrent tumor samples from all 8 IMA-resistance-GIST patients showed significantly increased KIT protein expression, even in the two patients with secondary mutations detected in their KIT gene; Secondly, down-regulation of KIT re-sensitized IMA-resistance-GISTs to Imatinib; Thirdly, KIT up-regulation likely increased overall KIT tyrosine kinase activity and stimulated growth of GISTs in the presence of Imatinib. These data suggest that the over-expression of KIT may play a major role in IMA-resistance and decreasing KIT expression or inhibiting the signaling pathways that regulate KIT may lead to significant clinical benefit for GIST patients with IMA-resistance [10, 11].

NF-κB is an ubiquitous transcription factor that is commonly activated in human malignancies [29, 30]. Previous studies have shown that the NF-κB signaling pathway plays a significant role in the regulation of KIT expression [25]. We showed that over-expression of p55PIK activated the NF-κB pathway, and led to increased KIT gene expression whereas p55PIK knockdown or blockade of p55PIK signaling with p55PIK inhibitor, TAT-N24, had the opposite effect on KIT protein expression. Pharmacological blockade of NF-κB by BAY11–7082 and the resultant decrease in KIT expression also led to Imatinib sensitivity in IMA-resistance-GIST. Our findings strongly suggest that NF-κB and/or its upstream p55PIK signaling pathway may be promising new therapeutic targets for the treatment of IMA-resistance-GIST patients. In this regard, NF-κB inhibitors may be effective in the treatment of IMA-resistance-GIST patients despite potential broader effects on other signaling pathways. It also should be pointed out that there likely are other signaling pathways that regulate the expression of KIT in GIST and these signaling pathways may contribute to the formation of IMA-resistance. Thus, it will be interesting to study the effects of targeting the p55PIK-NF-κB pathway as well as other pathways that regulate KIT expression as potential novel treatments for IMA-resistance in GIST patients.

The PI3K pathway is a major contributor to proliferation and survival in imatinib-sensitive and imatinib-resistant GIST [12, 31]. Recent studies showed that the combination of PI3K inhibitor and Imatinib treatment was more effective than PI3K inhibitor or Imatinib alone in GIST xenograft animal models [32]. The significant side effects and toxicity associated with currently available PI3K inhibitors and those in the drug development pipeline limit their clinical application. Thus, inhibiting specific PI3K isoform (isoform-specific PI3K inhibitors), including those of regulatory PI3K subunits, becomes an attractive alternative method to disrupt PI3K signaling. Recently, p55PIK-PI3K has emerged as a potential therapeutic target in cancer therapy [18, 19, 33]. p55PIK is one of the Class I_A_ PI3K regulatory subunits, and has significant sequence homology with PIK3R1 and PIK3R2 regulatory subunits in a proline-rich region and two Src homology 2 (SH2) domains [13]. However, it has an unique amino-terminus (N24) [15, 34] that enables PI3K to activate specific signaling pathways such as those involving Rb, PCNA, and NF-κB [18, 20, 35]. p55PIK protein expression is increased in several types of tumors and p55PIK-mediated signaling pathways play critical roles in cell cycle progression [15, 17, 19]. Furthermore, down-regulation of p55PIK by siRNA or its specific inhibitor (TAT-N24) inhibit tumor growth and progression by decreasing cell proliferation, inducing differentiation, and blocking angiogenesis [19, 20]. Here, we showed that increased p55PIK expression confers IMA-resistance in GIST cell lines, xenografts and GIST clinical specimens. The critical role of p55PIK in IMA-resistance was established by showing that a p55PIK-specific inhibitor, TAT-N24 as well as Lenti-shRNA^p55PIK^ restored Imatinib sensitivity in IMA-resistance-GIST cell lines and xenograft tumors. Our findings demonstrating the importance of p55PIK in IMA-resistance-GIST by its induction of KIT expression, raise the issue of how p55PIK expression is up-regulated in GIST during Imatinib treatment. Future research on the mechanism of p55PIK over-expression in IMA-resistance-GIST may help identify new strategies to prevent the development of IMA-resistance.

In summary, we have shown that p55PIK-PI3K activates NF-κB to increase the expression of KIT in IMA-resistance-GIST. Moreover, the p55PIK-NF-κB-KIT axis is an important pathway that is involved in IMA-resistance-GIST in clinical specimens. These findings strongly suggest that p55PIK and NF-κB might be effective therapeutic targets, in combination with Imatinib, for treating IMA-resistance-GIST and other malignancies associated with IMA-resistance. The over-expression of p55PIK and KIT as well as NF-κB phosphorylation also may be predictive biomarkers to detect GIST patients that likely have IMA-resistance.

## MATERIALS AND METHODS

### Cell lines and cell culture

GIST882 cells, a kind gift of Jonathan Fletcher (Dana-Farber Cancer Institute, Boston, MA), derived from a GIST patient with a homozygous missense mutation in KIT exon 13 (K642E) [12]. GIST-T1 cells, obtained from Biowit Technologies (Shenzhen, China), derived from a GIST patient with an in-frame deletion of 57 nucleotides in KIT exon 11 (V560Y579del) [21]. GIST882 and other cell lines used in this study were cultured at 37°C with 5% CO_2_ in DMEM supplemented with 10% fetal calf serum (FBS).

The primary culture GIST002 cells were established from an Imatinib-sensitive GIST patient and GIST005 cells from an Imatinib-resistant GIST patient (tumors reoccured 2 years after taking Imatinib). Fresh surgical GIST tissues were disaggregated with scalpels and incubated overnight at 37°C in a solution containing DNase and Collagenase B. Then the specimens were treated and cells were cultured as reported [22]. DNA sequencing of the successfully isolated GIST002 cells revealed a deletion in KIT exon 11 (del559), while in GIST005 cells there were mutations in KIT exon 11 (V559D) and KIT exon 13 (K642E).

### Establishment of IMA-resistance cell line, GIST882IR

GIST882 cells were inoculated in female nude mice. At day 7 after inoculation, mice were treated by oral gavage with Imatinib (100 mg/kg) daily for 3 weeks. Then, the tumors (named P1) were removed from mice, cut into small pieces and replanted to new female mice. Mice bearing tumors were treated with Imatinib (100 mg/kg) for 3 weeks. This procedure was repeated 6 times and tumors removed from the last passage (P6) were cut into small pieces and cultured in 6-well plates. The cells attached to the plates were expanded and examined for their response to Imatinib. The cells obtained were named as GIST882IR, as these cells were significantly resistance to Imatinib, in comparison to the parental GIST882 cells.

### Microarray analysis

RNA was extracted from cultured cells and tumor samples, the RNA integrity was checked using an Agilent 2100 Bio-analyzer. RNA samples were used as templates to make cDNA and analyzed for gene expression with Illumina Human HT12 v3 Expression Bead-Chips. The intensity of signal was quantified by using an Illumina Bead-Station 500GX Genetic Analysis Systems scanner.

### Construction of lentiviral systems expressing p55PIK and short hairpin RNA (shRNA) targeting p55PIK

A cDNA encoding human p55PIK (NCBI, NM_003629.3) was cloned into the lentivirus vector plasmid pCDF1 (System Biosciences, SBI). Lentivirus expressing p55PIK (Lenti-p55PIK) and control lentivirus (Lenti-con) were constructed and packaged following the manufacturer's instructions (System Biosciences, Wuhan).

Primers coding shRNA targeting p55PIK (5′-ggacuugcuuuaugggaaa-3′) and scramble primers coding a non-targeting RNA sequence were cloned into a lentivirus vector, GV115, derived from pENTR/U6 entry vector (Invitrogen). GV115 lentivirus vector also contained the cDNA sequence encoding GFP under the control of CMV promoter. Lentivirus expressing shRNA against p55PIK (Lenti-shRNA^p55PIK^) and control lentivirus (Lenti-sc) was then constructed and packaged, virus particles were titered using 293FT cells following manufacturer's instructions (Invitrogen).

### Construction of NF-κB reporter plasmid

For reconstruction of the pGL3-NF-κB element reporter system, the primers of NF-κB promoter element (sequence: 5′-gggactttccgggactttccgggactttcc-3′) were synthesized and cloned into the pGL-3 vector.

### Chemical compounds and biologic reagents

Imatinib (Glivec), obtained from Novartis, was dissolved in DMSO, and diluted to appropriate concentrations. BAY11–7082, obtained from Sigma, was dissolved in DMSO and stored at −20°C.

### Tumor samples from GIST patients

Primary and recurrent tumor specimens were obtained from GIST patients receiving surgeries and treated with Imatinib (400 mg/day) in in 3 hospitals at Wuhan city, Tongji Hospital, Renmin Hospital and Union Hospital for GIST. All patients were informed and provided written consent.

### Cell viability measurements

Cell viability studies were performed using a kit from Promega following manufacturer's instruction. All experimental points were measured in triplicates and were repeated at least three times.

### Mutation analysis

For mutation analysis, genomic DNA was isolated from tumor samples. Prior to collecting DNA, the tumor tissues were determined to contain more than 70% neoplastic cells by a pathologist.

The DNA region of full KIT coding sequences and intron–exon boundaries of the KIT exons 9, exons 11, exon 13, and exons 17, were screened for mutations. Primer sequences were used as below: exon 9: Forward: 5′-ggtcaccaaagtgcttattctt-3′; Reverse: 5′-gtgagtttgatgacagtatggtg-3′; exon 11 and exon 13: Forward: 5′-tgttctctctccagagtgctctaa-3′; Reverse: 5′-ctgggc tgttctaccccata-3′; exon 17: Forward: 5′-tgaacatcattcaagg cgta-3′; Reverse: 5′-tgttcagcataccatgcaaa-3′.

### Establishment of tumor xenograft model in mice

All animal experiments were conducted by following the Animal Study Guidelines of Huazhong University of Science and Technology. Cultured cells were resuspended in culture medium and 2 × 10^6^ cells (100 μl) were injected subcutaneously into athymic nude mice. At day 7 after inoculation, Imatinib was given by intragastric administration (100 mg/kg) as well as Lenti-sc or Lenti-shRNA^p55PIK^ (10^10^ tu/20 μl) was injected into tumors every 3 days [23]. As lentivirus vector contained the cDNA encoding GFP under the control of CMV promoter, the percentage of cells detecting GFP signals in tumor samples was measured by immunostaining using anti-GFP antibody and used as an indicator to show the lentivirus transfection efficiency of intratumoral injection. For this purpose, the number of GFP^+^ cells in 300 total cells was determined and the percentage of GFP^+^ cells was calculated.

In some experiments, at day 7 after inoculation Imatinib (100 mg/kg) was given by intragastric administration. In addition, TAT-N24 or control TAT-N24M fusion proteins (80 mg/kg) were injected into mice bearing tumors via tail vein every 2 days as described [19].

### RT-PCR and western blot

Total RNA was isolated using Trizol by following the manufacture's protocols, reverse transcription and RT-PCR was done as described [19].

Western blotting was done as described previously^16^. Antibodies to p55PIK, KIT, p65, pp65(Ser536), α-Tubulin, GAPDH were purchased from Santa Cruz Biotechnology.

### Statistical analysis

For xenograft experiments, tumor volume and weight measurements were expressed as mean ± standard error. Group differences in tumor volumes and weight were compared. *P*-values were calculated using an unpaired Student *t* test.

## SUPPLEMENTARY FIGURES AND TABLES


