# Supplementary material for: KIT over-expression by p55PIK-PI3K leads to Imatinib-resistance in patients with gastrointestinal stromal tumors
Source: Oncotarget. 2015 Oct 22;7(2):1367–79. doi: 10.18632/oncotarget.6011 (PMC4811466; doi:10.18632/oncotarget.6011)
Supplement: Supplementary file 1 [file oncotarget-07-1367-s001.pdf]

SUPPLEMENTARY FIGURES AND TABLE

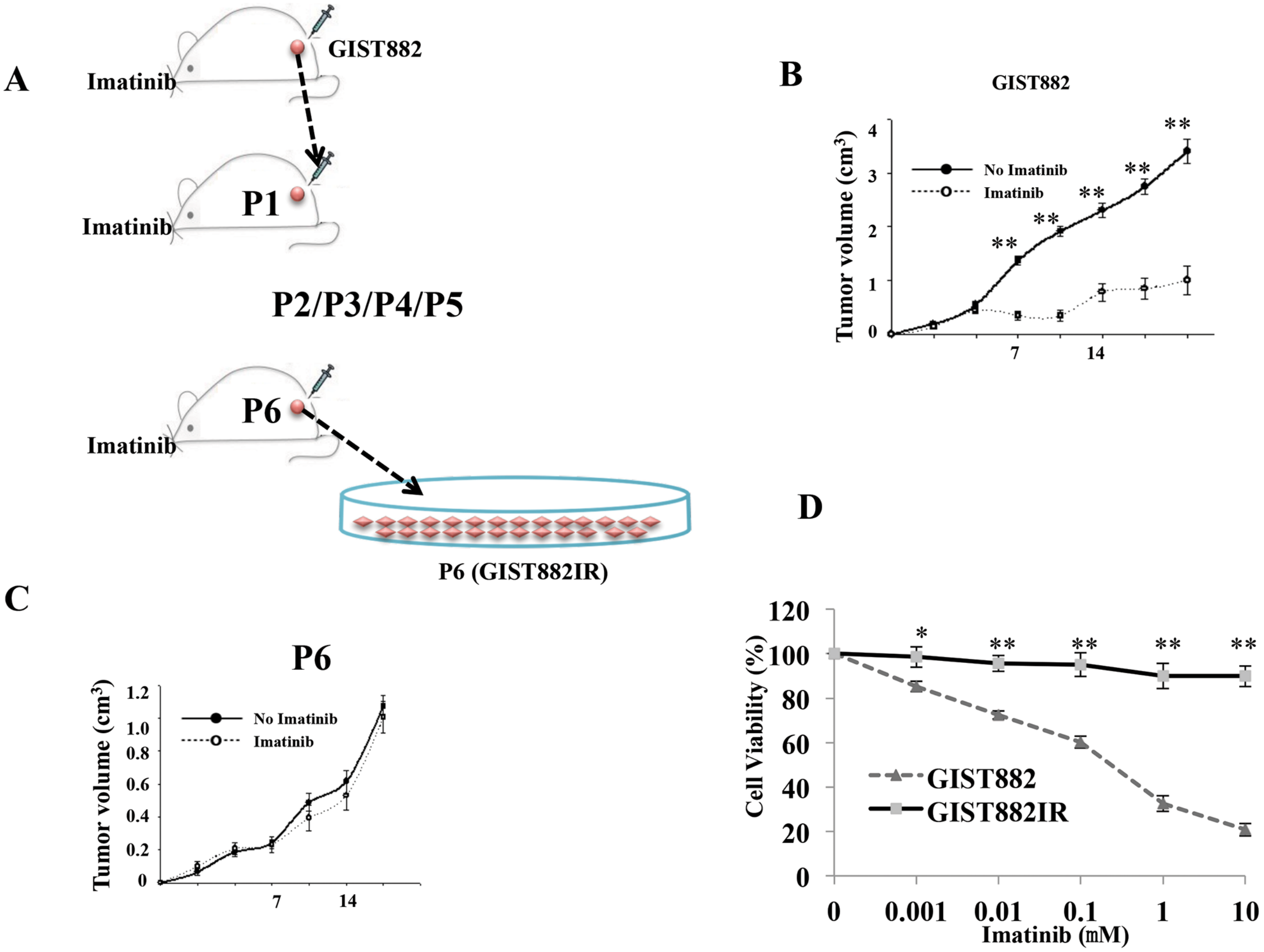

Supplementary Figure S1: Establishment of GIST882IR cells. A. GIST882 cells were inoculated in female nude mice and at day 7 after inoculation, mice were treated by oral gavage with Imatinib (100 mg/kg) daily for 3 weeks. Then, the tumors (named P1) were removed from mice, cut into small pieces and replanted to new female mice. This procedure was repeated 6 times and tumors removed from the last passage (P6) were characterized. **B.** Inhibitory effects of Imatinib on the growth of GIST882 cells *in vivo*. GIST882 cells were inoculated in nude mice and the mice were divided into two groups and administered with vehicle or Imatinib (100 mg/kg) daily for 3 weeks. The size of tumors was measured. $^{**}p < 0.01$. **C.** Growth curve of GIST882IR cells *in vivo* treated with or without Imatinib. Cells derived from P6 tumors were expanded and collected (named as GIST882IR). GIST882IR cells were inoculated in female nude mice and at day 7 after inoculation, mice were divided into 2 groups and treated by oral gavage either with Imatinib (100 mg/kg) or control solvent daily for 3 weeks. The tumor volumes were measured. **D.** IR in GIST882IR cells. Cell viability of GIST882 and GIST882IR cells after treatment with Imatinib was measured. $^{*}p < 0.05$; $^{**}p < 0.01$.

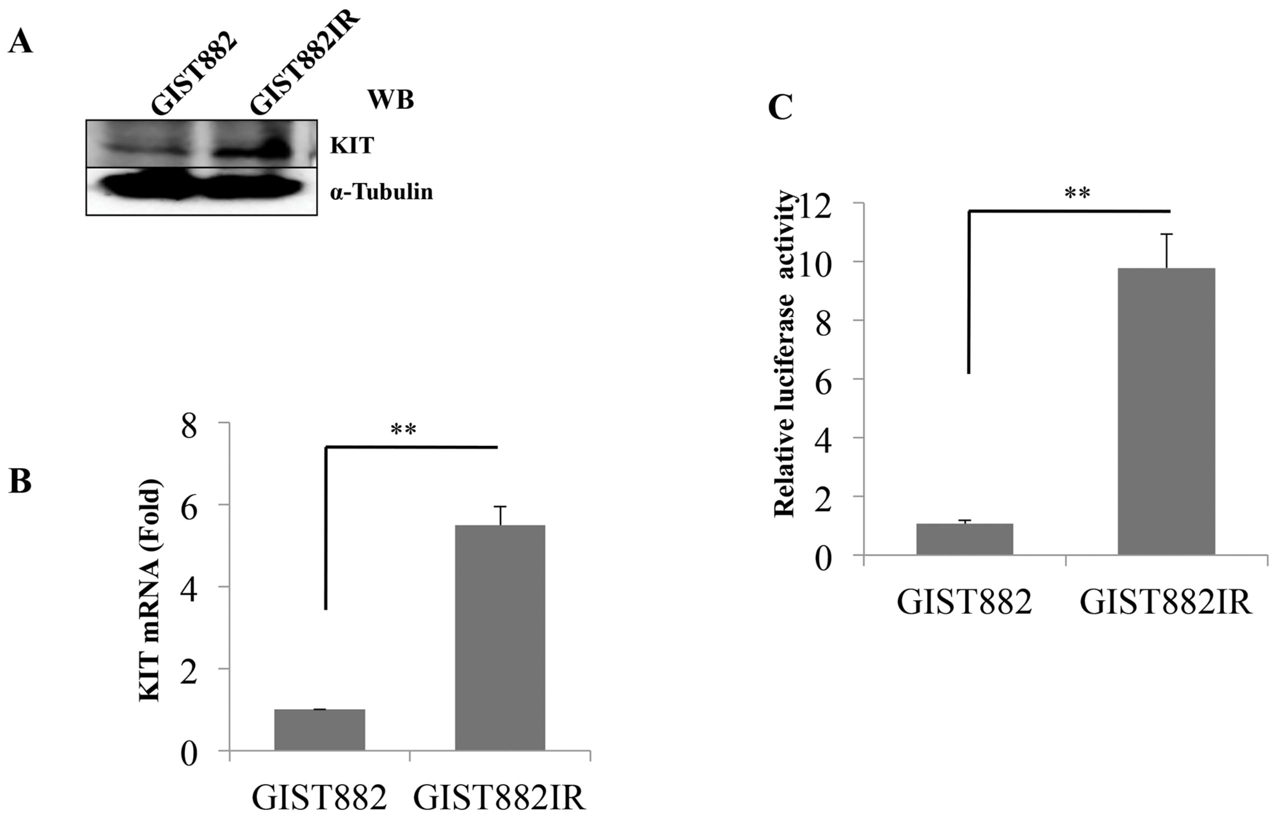

Supplementary Figure S2: Over-expression of KIT in GIST882IR cells. A. Protein level of KIT in GIST882 and GIST882IR cells. Lysates from GIST882 and GIST882IR cells were prepared and the protein levels of KIT were measured by Western blotting. **B.** mRNA level of KIT in GIST882 and GIST882IR cells. Total RNA was isolated from GIST882 and GIST882IR cells and the expression of KIT mRNA was determined. $**p < 0.01$. **C.** Transcription activity of KIT promoter in GIST882 and GIST882IR cells. Cultured GIST882 and GIST882IR cells were transfected with KIT promoter reporter plasmids, the cellular lysates were prepared 48 h after transfection and the luciferase activity was determined using a Kit from Promega. $**p < 0.01$.

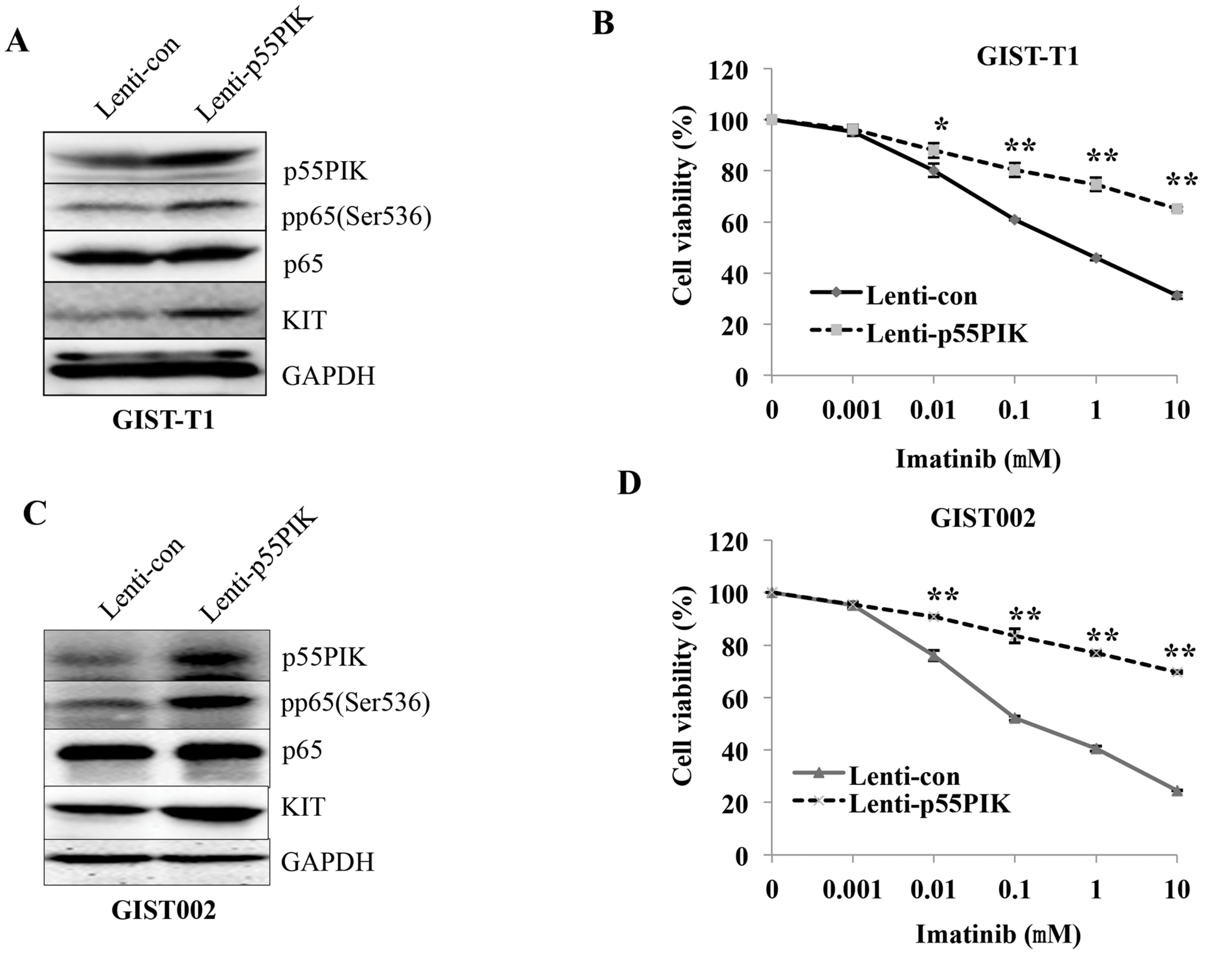

Supplementary Figure S3: Over-expression of p55PIK increased the expression of KIT and led to the IR in GIST-T1 and primary cultured GIST cells. A. Over-expression of p55PIK increased the expression of KIT and phosphorylation of NF-κB p65 in GIST-T1 cells. GIST-T1 cells were infected with Lenti-p55PIK or Lenti-con. Cell lysates were prepared and the expression of p65, pp65(Ser536), p55PIK, and KIT was examined by Western blotting. **B.** IR in GIST-T1 cells over-expressing p55PIK. GIST-T1 cells were infected with Lenti-p55PIK or Lenti-con overnight and treated with various concentration of Imatinib for 72 h. Cell viability was measured. **C.** Over-expression of p55PIK increased the expression of KIT and phosphorylation of NF-*03BA*B p65 in primary GIST002 cells. GIST002 cells were infected with Lenti-p55PIK or Lenti-con. Cell lysates were prepared and the expression of p65, pp65(Ser536), p55PIK, and KIT was examined by Western blotting. **D.** IR in primary GIST002 cells over-expressing p55PIK. GIST002 cells were infected with Lenti-p55PIK or Lenti-con overnight and treated with various concentration of Imatinib for 72 h. Cell viability was measured. *$p < 0.05$; **$p < 0.01$.

Supplementary Table S1: Characterization of tumor samples from GIST patients

No.	Sex	KIT mutations (primary tumors)	Duration between surgeries (Year)	KIT mutations (recurrent tumors)
1	Male	11^{V559D}	3.1	11^{V559D}
2	Female	$11^{V560del}$	2.7	$11^{V560del}$
3	Male	$11^{V559del}$	2.0	$11^{V559del}$, 13^{K642E}
4	Male	$9^{A502\text{-}Y503dup}$, $11^{K558V559del}$	1.1	$9^{A502\text{-}Y503dup}$, $11^{K558V559del}$
5	Male	11^{V559D}	2.5	11^{V559D}, 17^{Y823D}
6	Male	11^{W557R}	3.5	11^{W557R}
7	Male	$11^{W557D572del}$	2.2	$11^{W557D572del}$
8	Male	$11^{K558N+V559del}$	4.1	$11^{K558N+V559del}$
